# Supplementary material for: Content-rich biological network constructed by mining PubMed abstracts
Source: BMC Bioinformatics. 2004 Oct 8;5:147. doi: 10.1186/1471-2105-5-147 (PMC528731; doi:10.1186/1471-2105-5-147)
Supplement: Additional File 5 — The original Chilibot query results of the term "long-term potentiation (LTP)" and 22 other terms, limiting the latest references analyzed to the years 1990, 1995, 2000, and 2004. [file 1471-2105-5-147-S5.bz2 › chilibotAdditionalFile5/ltp2004/html/ERK_PI-3K.html]

 


 **ERK** and **PI-3K** 
  
Found 333 abstracts in PubMed,  **30 abstracts were retrieved and analyzed**.  


---

 Search Google  |
 PDF files only 
|  EDU domain only 

---

**Interactive relationship** (e.g. stimulation, inhibition, etc)

- Experiments based on inhibitors of specific signaling pathways, such as manumycin A, toxin A, U0126, PD98059 and wortmannin revealed that Ras, MEK and PI 3K  [ **PI-3K** ]  are involved in the activation of  **ERK** .  Ref: 14563359 Insect Biochem Mol Biol, 2003
- Assays with inhibitors and antibodies against proteins involved in signal transduction demonstrated that NAPS and TAPS elevated COX 2 expression via tyrosine kinase, src, PI 3 kinase  [ **PI-3K** ]  and PKC, followed by  **ERK**  activation.  Ref: 14708615 J Invest Dermatol, 2003

**Parallel relationship** (e.g. studied together, co-existance, homology, etc.)

- It has been reported that the activation of the extracellular signal regulated kinase 1 2  **Erk**  1 2 and phosphoinositide 3 PI3 kinase  [ **PI-3K** ]  plays a crucial role in the NGF induced differentiation of PC12 cells.  Ref: 14706634 Biochem Biophys Res Commun, 2004
- Thus, angiotensin II induces the phosphorylation of 4E BP1 via the PI 3 kinase  [ **PI-3K** ]  mTOR pathway, but not via  **ERK**  or p70 S6 kinase.  Ref: 14555183 Eur J Pharmacol, 2003
- The insulin induced suppression of IRS 2 mRNA and protein was blocked by the phosphatidylinositol PI 3 kinase  [ **PI-3K** ]  inhibitor, LY294002... PD098059.  Ref: 14596677 J Endocrinol, 2003
- The medfly hemocytes respond to LPS by changing their morphology, inducing the activation of several signaling pathways, including Ras MEK  **ERK** , PI 3K  [ **PI-3K** ]   **ERK**  and Rho pathways and contributing to LPS uptake.  Ref: 14563359 Insect Biochem Mol Biol, 2003
- C6 ceramide induces TrkA phosphorylation and selective activation of the phosphatidyl inositol 3 kinase PI3 kinase  [ **PI-3K** ]  Akt pathway but not the MAPK  **ERK**  pathway.  Ref: 14614956 Neuropharmacology, 2003
- Overall, DQ elicited the differentiation of PC12 cells through Trk A phosphorylation followed by  **Erk**  1 2 and PI3 kinase  [ **PI-3K** ]  activation.  Ref: 14706634 Biochem Biophys Res Commun, 2004
- KGF stimulates alveolar type II cell proliferation through the  **ERK**  and PI3 Kinase  [ **PI-3K** ]  pathways.  Ref: 14742297 Am J Respir Cell Mol BiolAm J Respir Cell Mol Biol, 2004
- This study showed that the effect of DQ on neurite outgrowth is mediated via the  **Erk**  1 2 and PI3 kinase  [ **PI-3K** ]  dependent pathways like NGF.  Ref: 14706634 Biochem Biophys Res Commun, 2004
- We investigated the role of the  **ERK**  p42 44 MAP kinase and PI3 kinase  [ **PI-3K** ]  pathways on alveolar type II cell proliferation and differentiation.  Ref: 14742297 Am J Respir Cell Mol BiolAm J Respir Cell Mol Biol, 2004
- Leptin increases cardiomyocyte hyperplasia via  **ERK**  and PI 3 kinase  [ **PI-3K** ]  dependent signaling pathways.  Ref: 14715711 EndocrinologyEndocrinology, 2004
- Calcium Sensing Receptor Induces Proliferation through p38 MAPK and PI3 kinase  [ **PI-3K** ]  but not  **ERK**  in a Model of Humoral Hypercalcemia of Malignancy HHM.  Ref: 14645111 EndocrinologyEndocrinology, 2004
- Application of either MAPK  **ERK**  or PI 3 kinase  [ **PI-3K** ]  pathway inhibitors disrupted VEGF induced angiogenesis, thereby indicating that both signaling pathways mediate this process.  Ref: 14663337 J Cereb Blood Flow Metab, 2003
- Persistence of signaling by the wild type AR in therapy resistant tumors is due to the increased receptor activity caused by cross talk of AR with multiple intracellular signaling cascades, especially the growth factor activated MAP kinase  **ERK**  and PI3 kinase  [ **PI-3K** ]  Akt pathways.  Ref: 14619959 Mol Cell Biochem, 2003
- We also aimed to determine if extracellular signal regulated kinase  **ERK**  and phosphatidylinositol 3 kinase PI3 kinase  [ **PI-3K** ]  are implicated in the action of SNAP.  Ref: 14656708 Am J Physiol Heart Circ PhysiolAm J Physiol Heart Circ Physiol, 2003
- Thus VEGF binding to the endothelial flk 1 receptor activates the MAPK  **ERK**  and PI 3 kinase  [ **PI-3K** ]  pathways, resulting in neoangiogenic events.  Ref: 14663337 J Cereb Blood Flow Metab, 2003
- These findings suggest that intracellular tyrosine kinases, MEK  **ERK**  and PI3 kinase  [ **PI-3K** ]  AKT pathways are involved in 5 S HETE stimulated pancreatic cancer cell proliferation but P38 kinase, JNK SAPK and PKC are not involved in this mitogenic effect.  Ref: 14707447 Oncology, 2003
- SUMMARY The angiogenic role of vascular endothelial growth factor VEGF receptors, flk 1 and flt 1, and their downstream signaling pathways, MAPK  **ERK**  and PI 3 kinase  [ **PI-3K** ] , were examined in a fetal rat cortical explant model after exposure to exogenous VEGF.  Ref: 14663337 J Cereb Blood Flow Metab, 2003
- Levels of phospho Akt and phospho  **ERK**  were greater in proximal truncated than in wild type G CSFR cells, suggesting that Gab2 is dissociated from PI 3 kinase  [ **PI-3K** ]  or  **ERK**  activities.  Ref: 14656892 BloodBlood, 2003
- Whether PI 3K  [ **PI-3K** ]  is an intermediate of Ras MEK  **ERK**  pathway or activates  **ERK**  via other signaling pathway it remains to be elucidated.  Ref: 14563359 Insect Biochem Mol Biol, 2003
- In addition, PI 3K  [ **PI-3K** ]  as well as Ras and MEK through  **ERK**  activation participate in LPS endocytosis.  Ref: 14563359 Insect Biochem Mol Biol, 2003
- Pretreatment with wortmannin or 2 4 Morpholinyl 8 phenyl 4H 1 benzopyran 4 one LY294002, a PI 3 kinase  [ **PI-3K** ]  inhibitor, suppressed angiotensin II induced phosphorylation, but a mitogen activated protein kinase MAPK extracellular signal regulated kinases  **ERK**  kinase 1 MEK 1 inhibitor, 2 Amino 3 methoxyflavone PD98059, and a p38 MAPK inhibitor, 4 4 Fluorophenyl 2 4 methylsulfinylphenyl 5 4 pyridyl 1H imidazole SB203580, had no effect.  Ref: 14555183 Eur J Pharmacol, 2003
- NF kappaB inhibitors N acetyl L cysteine and Bay 11 7085 and PI 3 kinase  [ **PI-3K** ]  inhibitor LY294002 inhibited the enhancing effects of IL 18, but MAPK p38 inhibitor SB203580,  **ERK**  inhibitor PD98059, and JNK inhibitor SP600125 did not.  Ref: 14872485 Arthritis Rheum, 2004
- Extracellular addition of ATP activated important cell survival proteins like PI 3 kinase  [ **PI-3K** ]  and extracellular regulated kinase  **ERK**  1 2.  Ref: 14761947 J Biol ChemJ Biol Chem, 2004
- This requires activation of the signaling enzymes Ras,  **Erk**  MAP kinase and PI3 kinase  [ **PI-3K** ] .  Ref: 14706690 J Physiol ParisJ Physiol Paris, 2004
